# A study on extracellular and cellular composition to advance towards the rational design of contractile human myocardium

**DOI:** 10.1016/j.mtbio.2026.103647

**Published:** 2026-09-04

**Authors:** Olalla Iglesias-García, Asier Ullate-Agote, Jiabin Qin, Aida Oliván-Viguera, Laura García-Mendívil, Gerardo Cedillo-Servin, Johannes Braig, Jose Valdés-Fernández, Inge Dokter, Ilazki Anaut-Lusar, Eduardo Larequi, Patxi San Martin-Uriz, Paula Aguirre-Ruiz, Pedro Vicente, Ricardo M. Rosales, Ana María Sánchez de la Nava, Ainitze Gereka Goienetxe, Ane Miren Zaldua, María Eugenia Fernández-Santos, Manuel García de Yébenes, Juan José Gavira, Miguel Castilho, Paula M. Alves, Margarida Serra, Ming Wu, Stefan Janssens, Tomasz Jüngst, Jürgen Groll, Jos Malda, Esther Pueyo, Manuel Doblaré, Joost P.G. Sluijter, Alain van Mil, Felipe Prósper, Manuel M. Mazo Vega

**Affiliations:** a Biomedical Engineering Program, Technological Innovation Division, CIMA Universidad de Navarra, Pamplona, 31008, Spain; b Instituto de Investigación Sanitaria de Navarra (IdiSNA), Pamplona, 31008, Spain; c Department of Cardiology, University Medical Center Utrecht, Laboratory of Experimental Cardiology, Regenerative Medicine Center Utrecht, Circulatory Health Research Center, University Utrecht, Utrecht, the Netherlands; d Aragón Institute for Engineering Research (I3A) University of Zaragoza, CIBER de Bioingeniería, Biomateriales y Nanomedicina (CIBER-BBN), Institute for Health Research Aragón (IIS Aragón), Zaragoza, 50018, Spain; e Biomaterial Engineering & Biofabrication, Department of Biomedical Engineering, Eindhoven University of Technology, Eindhoven, the Netherlands; f Department of Functional Materials in Medicine and Dentistry, Institute of Biofabrication and Functional Materials, University of Würzburg and KeyLab Polymers for Medicine of the Bavarian Polymer Institute (BPI), Pleicherwall 2, Würzburg, 97070, Germany; g Hemato-Oncology Program, Cancer Division, CIMA Universidad de Navarra, Pamplona, 31008, Spain; h iBET, Instituto de Biologia Experimental e Tecnológica, Apartado 12, Oeiras, 2781-901, Portugal; i Instituto de Tecnologia Química e Biológica António Xavier, Universidade de Nova de Lisboa, Av. da República, Oeiras, 2780-157, Portugal; j Department of Cardiology, Gregorio Marañón Health Research Institute (IiSGM), Hospital General Universitario Gregorio Marañón, Madrid, 28007, Spain; k Center for Biomedical Research in Cardiovascular Disease Network (CIBERCV), Madrid, 28029, Spain; l Leartiker S. Coop., Makina-Xemein, 48270, Spain; m Department of Cardiology, Clínica Universidad de Navarra, Madrid, 28027, Spain; n Department of Cardiology, Clínica Universidad de Navarra, Pamplona, 31008, Spain; o Institute for Complex Molecular Systems, Eindhoven University of Technology, the Netherlands; p Department of Cardiovascular Sciences, KU Leuven, Leuven, Belgium; q Department of Cardiovascular Medicine, University Hospitals Leuven, Leuven, Belgium; r Department of Orthopedics, Regenerative Medicine Center Utrecht, University Medical Center Utrecht, Utrecht University, Heidelberglaan 100, Utrecht, 3584 CX, the Netherlands; s Department of Clinical Sciences, Faculty of Veterinary Medicine, Utrecht University, Yalelaan 8, Utrecht, 3584 CM, the Netherlands; t Hematology and Cell Therapy, CCUN, Clínica Universidad de Navarra, Pamplona, 31008, Spain; u Centro de Investigación Biomédica en Red Cáncer (CIBERONC), Madrid, Spain

**Keywords:** Cardiac tissue engineering, hiPSC, Melt electrowriting, Biopolymer scaffold, Hydrogel, Next-generation sequencing, Local microenvironment

## Abstract

The recapitulation of the physiological cellular composition, 3D structure and mechanics of the human myocardium is key to improving the biofabrication of cardiac tissues. To advance the development of engineered heart patches, with significant potential for human cardiac repair, we assessed the impact of their cellular and extracellular constituents on tissue organization and function, by using advanced biofabrication and next-generation sequencing technologies. Combining melt electrowriting (MEW) fibrillary scaffolds with human induced pluripotent stem cell (hiPSC)-derived cardiomyocytes (hiPSC-CMs) and cardiac fibroblasts (-CFs), we generated human engineered cardiac tissues (MEW-hECTs) by casting in two different biomaterial compositions (fibrin and gelatin-methacryloyl (GelMA)), and varying proportions of the cardiac constituent cells. Under the conditions tested, fibrin-hECTs displayed improved tissue formation, coordinated contraction, structural organization, and electrophysiological behavior compared with GelMA-hECTs. Transcriptomics analysis indicated that fibrin-hECTs exhibited an increase in maturation-associated gene expression signatures compared with GelMA-hECTs, whereas a longer remodeling process of the synthetic environment was required in GelMA. Surprisingly, within the investigated MEW-based composite system, the inclusion of CFs had no positive impact on tissue organization and impaired the electrophysiological properties of myocardial constructs, increasing susceptibility to arrhythmias in computational simulations calibrated with experimental electrophysiological data. This information will help devise advanced myocardial tissues by enabling a comprehensive assessment of the main components, ultimately reflecting the unique native cardiac 3D organization.

## Highlights

• Multidimensional analysis identifies hydrogel composition as a key determinant of MEW-hECTs performance.
• Fibrin but not GelMA in MEW-based composites supports improved cardiac tissue formation and maturation-associated features.
• Adding hiPSC-CFs hinders tissue organization and electrophysiological behavior in MEW-hECTs.
• hiPSC-CFs increase functional disruption and *in silico* arrhythmogenic susceptibility in MEW-hECTs.

## Graphical abstract

This study presents an integrated MEW-based platform combining multimodal characterization to systematically evaluate how biomaterial and cellular composition influence the organization and function of engineered human cardiac tissues, providing a foundation for the development of highly biomimetic myocardium with strong translational potential for heart repair.

**Figure:**
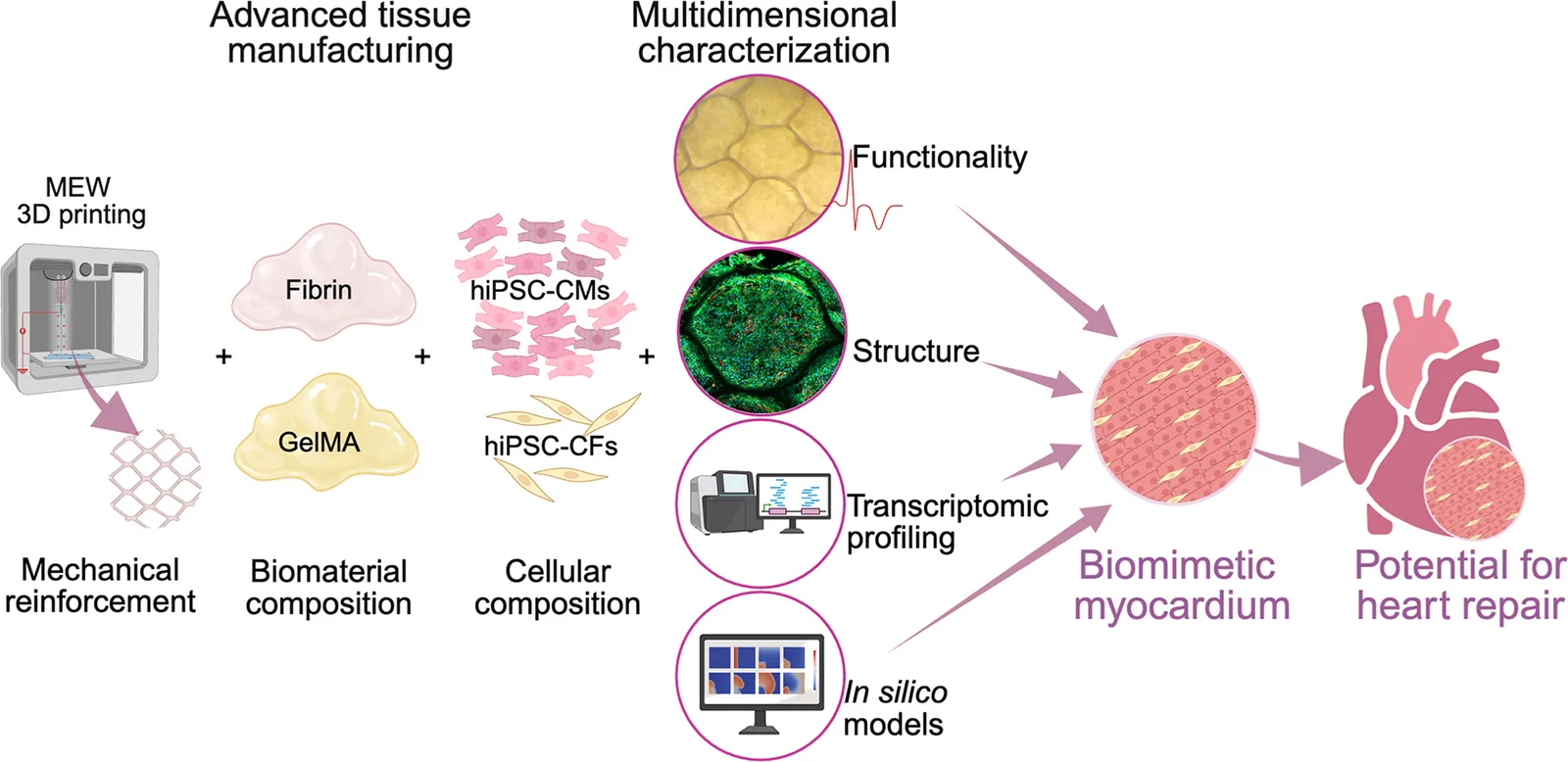

## 1 Introduction

Cardiac repair using engineered myocardium is steadily becoming a clinical reality. Almost 20 years after the initial report on human induced pluripotent stem cells (hiPSC) [1], the fabrication of an engineered heart patch produced from hiPSC-derived heart muscle cells has led to the first successful applications in patients with advanced heart failure [2,3]. After 25 years of pre-clinical development [4,5], it has now been demonstrated in rhesus macaques that implanted heart patches, consisting of up to 200 million human cells embedded in a collagen hydrogel, led to an improvement in cardiac function through remuscularization of the damaged heart, without significant side effects. The study also presented the first clinical case report, confirming remuscularization of a patient's heart following transplantation with 10 patches, composed of 400 million cells total [2]. Building on these findings, a subsequent phase 1-2 clinical trial has recently demonstrated the feasibility of this approach at higher doses, with implantation of up to 20 patches per patient (corresponding to 800 million cells), and provided further evidence supporting its safe and effective application for remuscularization of the human heart [6]. This investigation represents a major breakthrough, paving the way for heart failure treatment using human engineered cardiac tissue (hECT). Moving forward, the evolution of human cardiac repair and tissue regeneration using biofabrication depends on advancing technologies that allow the fabrication of hECTs with enhanced biomimicry capable of producing significant contraction, efficient force generation to support myocardial function, and electrical synchrony (no induction of arrhythmias). However, accurately replicating the complex architecture and functional hallmarks of the human heart remains a major challenge.

Despite substantial progress in cardiac tissue engineering using diverse fabrication techniques, cell types and biomaterials, the generation of 3D tissues that truly reproduce the physiological properties of the native human myocardium has not yet been fully achieved [7]. Recent efforts have focused on fabricating chambered cardiac constructs that recapitulate the heart's geometrical complexity [[8], [9], [10], [11]]. Nevertheless, the technology remains limited, and further studies are needed to evaluate long-term tissue functionality. Hydrogels are amongst the most widely studied type of biomaterials in cardiac tissue engineering. Fibrin, in particular, has been extensively used to generate hECTs due to its high biocompatibility and extracellular matrix (ECM)-like properties (including high elasticity, viscosity and cell-interaction capabilities), typically by casting cell-hydrogel suspensions in molds placed between or around flexible posts. Notably, the generation of hECTs with the highest level of maturation to date has been achieved through the application of electrical stimulation to fibrin-based constructs [12]. In addition, progress has also been made in generating clinical-size cardiac tissues using fibrin hydrogels, representing an important step toward therapeutic applications [13,14].

Synthetic biomaterials have also been employed in cardiac tissue engineering [15,16]. Among them, gelatin methacryloyl (GelMA) has gained significant traction in tissue engineering due to its biocompatibility, biodegradability and highly tunable properties [17]. However, in contrast to collagen or fibrin, GelMA does not yet have such extensive development directed towards clinical translation [[18], [19], [20], [21], [22]], though some groups have integrated GelMA into composite systems to better mimic the mechanical properties of native myocardium. For example, Lee et al. fabricated hybrid scaffolds using GelMA and conductive nanoparticles as cardiac patches capable of providing a more *in vivo*-like microenvironment, guiding maturation of cardiac tissues [20]. The Brenda Ogle group developed a bioink formulation by combining GelMA with collagen methacrylate (ColMA), fibronectin and laminin-III, that allowed hiPSC expansion and subsequent *in situ* cardiac differentiation, ultimately enabling fabrication of a miniature human heart chamber [23]. Furthermore, GelMA has also been combined with collagen to enhance cellular rearrangement and connectivity, obtaining contractile cardiac constructs [24].

In parallel, advanced biomaterial fabrication techniques have been developed to generate complex, hierarchical cell-material constructs [25]. A promising one is melt electrowriting (MEW), an additive fabrication technique capable of depositing micro-to-nano scale fibers with highly controllable architectures [26]. Previous studies have demonstrated the feasibility of using MEW to engineer myocardial tissues and the influence of the microarchitecture in cellular maturity and organization [24,[27], [28], [29]]. More recently, the exceptional capabilities of this technology were demonstrated by generating fiber scaffolds with diamond-shaped pores that accurately mimic the in-plane contraction of the human myocardial layers, allowing the fabrication of superior human cardiac tissues with therapeutic effectiveness [30].

Overall, these studies have demonstrated significant advantages in using advanced manufacturing technologies to generate more biomimetic human myocardium in the laboratory. However, reproducing the natural composition and functional properties of the adult heart, such as proper cellular arrangement, remains a challenge and are crucial aspects for high-fidelity tissue manufacturing. The human heart relies on a complex ensemble of specialized cardiovascular cells to maintain and support its function. While progress has been made in modelling the cellular composition of the adult myocardium, including testing different cell-type ratios to identify the most functional combinations [31], the way these biological components dictate hECT structure and function is not yet fully understood. Advances in transcriptomic technologies have provided powerful tools to track changes in cellular signaling during human heart development [[32], [33], [34]]. Previous studies have reported the use of this approach to analyze the maturation of engineered heart tissues [5,30,35,36], yet again, its systematic application to characterize and compare different engineered tissue designs in-depth might be key to guide the creation of more advanced tissues.

In the present work, we first compare two of the most widely used hydrogels in tissue engineering (fibrin and GelMA) as reinforcements for MEW scaffolds, and secondly, investigate the effect of modulating the cardiac cellular composition (100:0, 95:5, 90:10 and 80:20 of CM:CF) to generate optimal functioning and structured MEW-hECTs with higher therapeutic potential. MEW-hECTs were extensively characterized via imaging, structural analysis, and metabolic, electrophysiological and transcriptomic profiling. The use of fibrin exhibited superior biocompatibility and improved contractile, structural and calcium handling properties compared to GelMA. Transcriptomic profiling using bulk RNA-seq demonstrated an increase in maturation-associated gene signatures for fibrin-hECTs. Finally, our results show that, for MEW-hECTs, the inclusion of hiPSC-CFs does not improve tissue development, impairing the electrical properties of the myocardial constructs and even increasing propensity to arrhythmias.

## 2 Materials and methods

### 2.1 Melt electrowriting (MEW)

Printing of MEW scaffolds was performed as previously described [37]. In brief, MEW scaffolds were generated by printing polycaprolactone (PCL, Corbion, The Netherlands) on a 1 mm thick glass slide using a Discovery1 bioprinter system (RegenHU, Switzerland). PCL was heated to 80°C and extruded through a 24 G nozzle. Custom G-code was used to control the tool head. A voltage range of 6 kV, a pressure of 0.2 MPa, a collector distance of 3-4 mm and a trapezoidal velocity profile with a speed range of 6-8 mm/s were applied. Scaffolds were printed with a thickness of 35 layers to compare fibrin and GelMA hydrogels and 20 layers to analyze cell proportions (350 and 200 μm thick, respectively). After printing, the scaffolds were sprayed with 70% ethanol and gently released from the glass slide. Any excess ethanol was allowed to evaporate.

### 2.2 hiPSC culture and differentiation

All experiments were performed using the ESi007-A hiPSC line. Cells were maintained in mTESR™1 medium (Stem Cell Technologies) on growth factor reduced matrigel (GFR-MG) coated plastic surfaces (1:80 dilution), and passaged 1:20 by incubation with 0.5 mM EDTA (Invitrogen) every 4-5 days. Differentiation towards the cardiac lineage was performed as previously described [38]. Human iPSC-CM differentiation was induced using a biphasic modulation of the Wnt pathway. Lactate purification was performed once cells acquired spontaneous contraction (day 9-10) by culturing them for two 72-h rounds in RPMI no glucose supplemented with 4 mM lactate. Between the two cycles in lactate, the cells were replated, after incubation with TrypLE (Gibco) for 7-10 min at 37°C. Human iPSC-CMs were maintained in RPMI supplemented with B27 (RPMI B27) with frequent media changes and used after the second round of lactate purification. hiPSC-CMs were characterized by flow cytometry for cTNT and immunofluorescence staining for α-actinin. Human iPSCs were differentiated into CFs using an established protocol [39], where Wnt is first activated and cells are directed towards the epicardial lineage and CF specification. After their differentiation, cells were kept in culture in FibroGro™-LS medium (Millipore) supplemented with 10 μM SB431542, 10 ng/mL FGF2, and 1% P/S until their use. Cells were split 1:3 by incubation with TrypLE for 5 min at 37°C and used between passages 1-2 for experiments in this study. Human iPSC-CFs were characterized by flow cytometry and immunofluorescence staining for DDR2.

### 2.3 Tissue generation

Prior to cell seeding, scaffolds were cut with a punch biopsy of 8 mm (KAI MEDICAL) and treated with O_2_ plasma (Diener Electronic) for 5 min, in order to increase their hydrophilicity and facilitate infiltration of the cellular suspension. The scaffolds were then incubated in 70% ethanol during 30 min, washed three times in sterile distilled water and left to dry. Scaffolds were seeded with 6 × 10^4^ cells per μL of hydrogel. First, a tissue mastermix was made of cells mixed with RPMI B27 media supplemented with 10% KSR, 1% P/S and 10 μM Y27. To generate GelMA-hECTs, cells were resuspended in 45 μL of mastermix with 5% GelMA, pipetted into a Teflon mold where the MEW scaffolds were placed and crosslinked for 45 s using Ru/SPS under visible light irradiation. Same volume of tissue mastermix plus 6 mg/mL bovine fibrinogen (Sigma Aldrich) and 1.5 μL thrombin (100U/mL (Biopur)) were used to generate fibrin-hECTs as described in Ref. [40]. Samples were transferred to culture plates with RPMI B27 supplemented with 10% KSR, 1% P/S, and 10 μM Y27. On the next day, media was refreshed with RPMI B27 without Y27. For fibrin-based tissues, the media was supplemented with aprotinin (0.1% (wt/vol); 33 μg/mL) (Sigma Aldrich) to avoid fibrin degradation. Medium was changed every other day from there on.

### 2.4 Alamar Blue assay

Cell metabolic activity was assessed using the Alamar Blue assay for cell viability (Invitrogen), following manufacturer's instructions. Briefly, Alamar Blue solution was added to phenol red-free media (Life Technologies) to a final concentration of 10% (v/v), and constructs were incubated in this solution for 4 h at 37°C in 12-well plates. 100 μL of the supernatant was then collected, transferred to a 96-well plate, and fluorescence emission was measured in a plate reader (BMG Labtech).

### 2.5 RNA extraction, RT, qPCR

Total RNA was isolated from differentiated cells and cardiac tissues using Trizol RNA Isolation Reagent (Life Technologies), following the manufacturer's instructions and 1 μg RNA was next retrotranscribed into cDNA using the Prime Script RT Reagent Kit (Takara). Quantitative real time PCR was performed with 5 ng of cDNA in a PowerUp™ SYBR™ Green Master Mix (Applied Biosystems) and carried out in a QuantStudio 5 Fast Real-Time PCR system (Applied Biosystems). Human GAPDH was used as housekeeping gene and results analyzed using the 2^−ΔΔCt^ method.

### 2.6 Bulk RNA-seq library preparation and sequencing

Roughly 150 ng of high-quality total RNA (RIN > 8) were used for transcriptomic interrogation of (i) GelMA and fibrin-hECTs at 4 weeks post-generation using hiPSC-CMs and -CFs at a 9:1 ratio (N = 3 per condition) and (ii) fibrin-hECT at 4 weeks generated with 0, 5, 10 and 20% hiPSC-CFs (N = 3 per condition). For the former, Illumina's Stranded Total RNA Prep Ligation with Ribo-Zero Plus was used according to the manufacturer's instructions. Briefly, cytoplasmic and mitochondrial rRNAs as well as beta globin transcripts were depleted from the samples. The remaining RNA was fragmented and reverse-transcribed. A second strand cDNA synthesis step removed the RNA template while incorporating dUTP in place of dTTP in order to preserve strand specificity. Next, double-stranded cDNA was A-tailed, then ligated to Illumina anchors bearing T-overhangs. For the latter, Illumina's Stranded mRNA Prep Ligation was used according to the manufacturer's instructions. Briefly, oligo(dT) magnetic beads were used to capture and purify poly-adenylated mRNA molecules from total RNA. The purified mRNA was then fragmented and reverse-transcribed to cDNA using random primers.

In both cases, a second strand cDNA synthesis step removed the RNA template while incorporating dUTP in place of dTTP in order to preserve strand specificity. Next, double-stranded cDNA was A-tailed, then ligated to Illumina anchors bearing T-overhangs. PCR-amplification of the library allowed the barcoding of the samples with 10 bp dual indexes and the completion of Illumina sequences for cluster generation. Libraries were quantified with Qubit dsDNA HS Assay Kit (Thermo Fisher, Oregon, USA) and their profile was examined using Agilent's HS D1000 ScreenTape Assay (Agilent, CA, USA). Sequencing was carried out in an Illumina NextSeq2000 using paired-end, dual-index sequencing (Read 1: 59 cycles; i7: 10 cycles; i5: 10 cycles Read 2: 59 cycles) at a depth of either 30 or 50 million reads per sample for total RNA or polyA mRNA respectively.

### 2.7 Bulk RNA-seq analysis

Samples were demultiplexed using Illumina *bcl2fastq* software (v.2.20.0), reads trimmed and quality filtered with *Trim Galore* (v0.6.6) and aligned to the human genome (GRCh38) with *STAR* (V2.7.0d) using default parameters. Gene expression quantification was performed using the *featureCounts* function implemented in the R package *Rsubread* (v2.4.3) counting uniquely mapped reads with reverse strandness. The Ensembl v103 gene annotation was considered as the reference. Downstream analyses were performed with R v4.4.0. The *filterbyExpr* function implemented in the *edgeR* package (v4.4.2) was used to filter out genes with low number of counts for downstream analyses. In addition, only protein coding genes were kept for the ECM-mimicking hydrogels comparison. Data normalization, transformation (considering variance stabilizing transformation), principal component analysis and differential expression analyses with the Wald test and an FDR of 0.05 were performed with the *DESeq2* package (v1.46.1). The effect of the differentiation was included in the experimental design for the hiPSC-CFs proportion comparison. Overrepresentation analyses (ORA) of the differentially expressed genes (shrunken log2 fold-change with the lfcShrink function > 0.58) were performed with enrichR (v3.4) against the “GO_Biological_Process_2025” database. Gene Set Enrichment analysis (GSEA) was performed with *fgsea* (v1.32.4), ranking genes by the shrunken log2 fold-change and considering the Gene Ontology Biological Processes (GOBP) database. The resulting p-values were corrected with Benjamini-Hochberg, setting a 0.05 cutoff for significance. The gene sets were downloaded with msigdbr (v 7.4.1). Selected processes were plotted with the plotEnrichment function. Finally, to evaluate the impact of hiPSC-CFs,we considered all the DEG (with an FDR < 0.05) of the 100% hiPSC-CMs condition against each hiPSC-CFs proportion condition as input for the degPatterns function from the library DEGreport (v1.42.0) to look for different expression patterns using regularized log-transformed data, obtained with the rlog function. ORA of each gene pattern was performed with enrichR.

### 2.8 Comparison of GelMA and fibrin-hECTs with *in vivo* data

Bulk RNA-seq sequencing data from heart samples at different developmental stages, including adult, from published studies [[41], [42], [43], [44], [45], [46]] was retrieved from public repositories (NCBI GEO and ArrayExpress). Library processing (quality control, alignment and gene expression quantification) was the same as for our samples to ensure consistency, only including necessary modifications if they were single-end or unstranded. Any diseased sample, as well as those for which the predicted sex differed from the informed one, were discarded (see Supplementary Table 1 for sample information). Non protein-coding genes and histones were filtered out, to better compare the polyA mRNA libraries from the *in vivo* studies with our total RNA data. In addition, we removed genes with low number of counts for downstream analyses. Data normalization, transformation (considering variance stabilizing transformation) and principal component analysis were performed with the *DESeq2* package. Gene set scores per sample were obtained using the gsva function from the GSVA package (v2.0.7) for the vst counts.

### 2.9 Live/dead staining

Cell survival was assessed using the Live/Dead Viability/Cytotoxicity kit (Invitrogen) following manufacturer's instructions. Calcein AM and ethidium homodimer were both diluted 1/500 in RPMI B27, added to samples and incubated for 30 min at 37°C, after which media were changed and samples imaged in a Thunder microscope (Leica Microsystems) at 10x magnification and analyzed using ImageJ software.

### 2.10 Immunostaining analysis

For staining, engineered cardiac tissues were fixed with Zinc buffered formalin (Thermo Scientific) for 30 min at RT, followed by three washes in 1X PBS and stored at 4°C until use. Samples were incubated in blocking solution (1X PBS and 3% BSA (Sigma Aldrich)) for 20 min at RT after 0.2% Triton-X100 (Sigma Aldrich) permeabilization. Samples were stained overnight at 4°C with anti-cardiac α-actinin (1:400, Sigma Aldrich, A7811) and anti-vimentin (1:500, Abcam, ab92547) primary antibodies diluted in 1% BSA (Sigma Aldrich). After 3 washes in 1X PBS, samples were incubated with Alexa Fluor 488- and 594-conjugated secondary antibodies (1:500, Invitrogen) diluted in 1% BSA for 1 h at RT. Finally, samples were washed thrice with 1X PBS and incubated with DAPI (1:500, Sigma Aldrich, D9542) diluted in 1X PBS for 30 min at RT. Images were recorded using a LSM 800 Zeiss Confocal microscope, and analyzed using ImageJ software. Cardiomyocyte apoptosis was assessed using the TUNEL assay kit (11684817910, Roche), following the manufacturer's directions. Images were captured at 40x magnification, and the percentage of apoptotic nuclei was determined by dividing the number of TUNEL-positive nuclei by the total number of nuclei per image.

### 2.11 Quantification of Z-band spacing and filament alignment

Alpha-actinin confocal stacks from 3D samples were used for quantifying sarcomere length and orientation. A custom macro was developed based on ImageJ (available upon request). A minimum of 20 and maximum of 40 sarcomeres were analyzed per plane, with interplane separation of at least 10 μm. Specifically, at least 150 sarcomeres were quantified (n ≥ 150) per sample for each independent experiment (N = 3; n = 3). User was blinded to the group of origin to avoid any bias in the analysis.

### 2.12 Histology

Fixed MEW-hECTs were included in 2% agarose before paraffin inclusion. Thin sections (5 μm) were cut every 0.5 mm of depth and stained in Ehrlich's hematoxylin (Sigma). Bright-field digital images were acquired with an Aperio CS2 scanner (Leica Biosystems, Nussloch, Germany) and images processed with the ImageScope software (Leica Biosystems). Histological preparations were quantified using ImageJ software. Images were imported into Adobe Photoshop and formatted.

### 2.13 Optical mapping

High temporal and spatial resolution videos of transmembrane voltage and CaT activity in the engineered cardiac tissues were recorded by optical mapping with a MiCAM O5-Ultima CMOS camera (SciMedia, Costa Mesa, CA). Tissues were stained by immersion in culture medium supplemented with RH237 (voltage-sensitive dye, Invitrogen, Carlsbad, CA) at 10 μm for 15 min and with Rhod-2 AM (calcium-sensitive dye, Invitrogen, Eugene, OR) at 5 μm for 30 min under incubation conditions (37°C, 5% CO2). Blebbistatin (10 μm, Tocris Bioscience, St. Louis, MO) incubation during 30 min prevented motion artefacts. Following the incubation with the dyes, the tissues were placed in a heated chamber (RC-27NE in a PM-6 heated platform, Warner Instruments) at 36.5°C with 1 mL pre-oxygenated Tyrode's solution (supplemented with 30 mM BDM to prevent motion artefacts). Both voltage and calcium activities were mapped using a sampling frequency of 500 Hz and a spatial resolution of 100x100 pixels per frame, with a field of view of 3.1 mm. The tissues were field-stimulated with two platinum electrodes using an A385 stimulator (World Precision Instruments, Sarasota, FL) to apply monopolar pulses of 70 mA amplitude and 3 ms duration. After a short period of stimulation to allow the tissues to adjust to the pacing rate, 20-40 s recordings were acquired at pacing frequencies of 0.25, 0.5, 1, 2, 3 and 4 Hz.

### 2.14 Modelling and simulation

Computational modeling and simulation were employed to evaluate the impact of CF inclusion on the electrical activity of hiPSC-CMs within hECTs. *In silico* hECT models incorporated scaffolds with hexagonal pore geometries [47], consistent with experimental data. A baseline hECT model containing only hiPSC-CMs was first constructed, in which the Paci cellular AP model [48] was applied to all mesh nodes to represent CM electrophysiology. Electrical propagation was described using the monodomain model, and the longitudinal diffusion coefficient was adjusted to 0.000036 cm^2^/ms to reproduce the mean CV measured experimentally. For models including CFs, selected mesh nodes were assigned the MacCannell AP model of CFs [49], with CM:CF ratios of 95:5, 90:10, and 80:20, corresponding to experimental conditions. The longitudinal diffusion coefficient for CFs was set to 0.000003 cm^2^/ms, ensuring that the CV in the 80:20 model matched the experimental mean CV. The transverse-to-longitudinal conductivity ratio was fixed at 0.25 in all simulations.

CV and the repolarization time gradient (RTG) were calculated as described previously [47]. Spiral waves were induced using an S1-S2 cross stimulation protocol, and the vulnerability window (VW) length was defined as the time window during which a premature stimulus can elicit reentry [50].

All simulations were performed using ELECTRA, an in-house cardiac electrophysiology solver [51,52]. Temporal integration was implemented using a dual adaptive time integration algorithm based on the Forward Euler method [53], while spatial derivatives were numerically integrated via the Finite Element Method.

### 2.15 Statistical analysis

Data is expressed as mean ± SEM. Normal distribution of data was tested using the Shapiro-Wilk and Kolmogorov-Smirnov normality tests. Comparisons between experiments involving two groups were performed using Student's *t*-test. For experiments involving more than two groups, data were analyzed using 1-way ANOVA followed by Tukey's HSD or Kruskal-Wallis test for non-parametric analyses, and 2-way ANOVA followed by either Tukey's HSD or Šidák correction test. Analysis were performed using GraphPad Prism 9 (GraphPad Software Inc.), and differences were considered significant when p < 0.05.

## 3 Results

### 3.1 Fibrin supports the formation and functionality of MEW-based engineered cardiac tissues

**Fig. 1:**
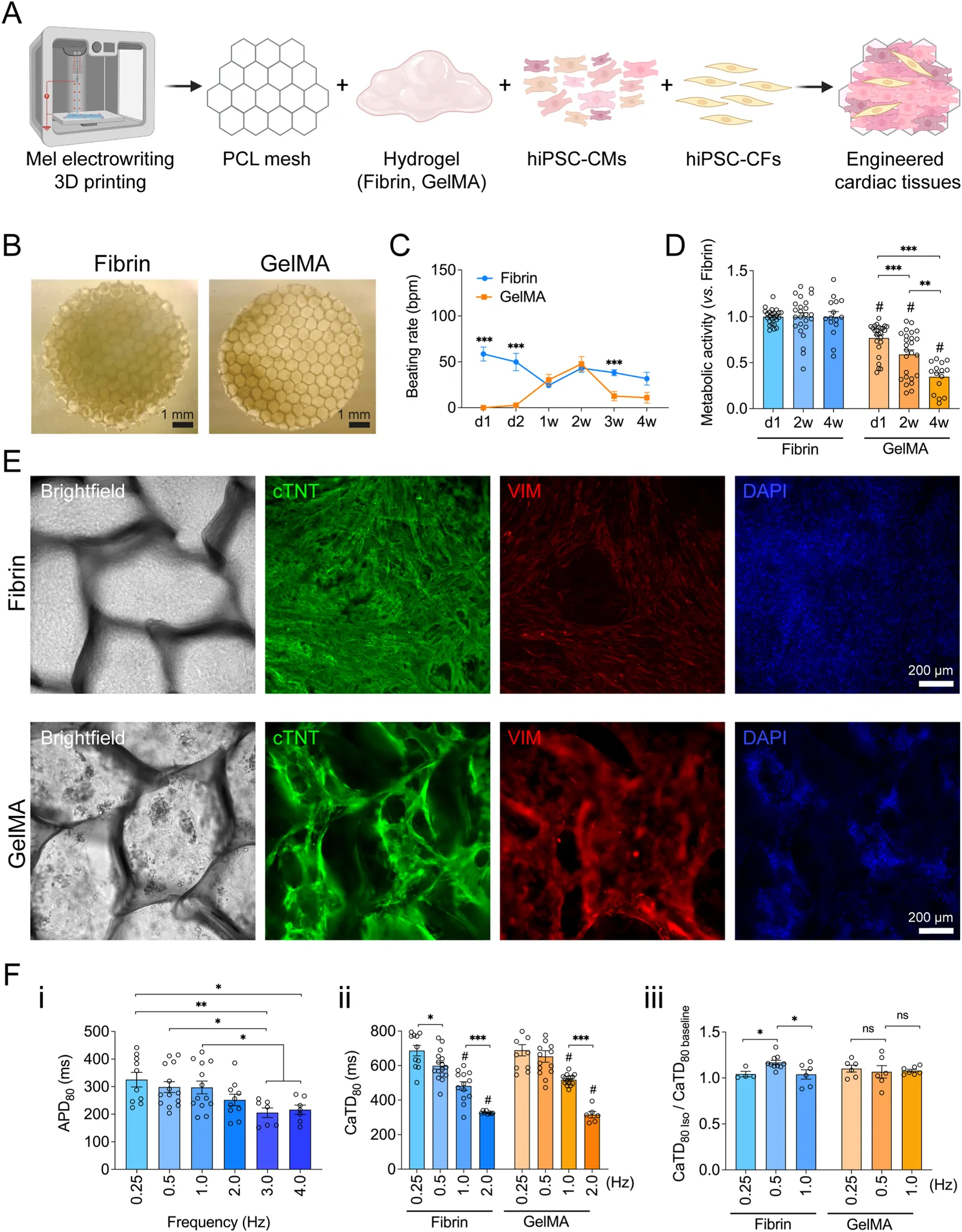
hECT formation capacity using fibrin and GelMA hydrogels combined with MEW-Scaffolds. A) Scheme of MEW-based engineered tissue fabrication (made with BioRender). B) Representative images of fibrin- and GelMA-hECTs 1 week after tissue formation. C) Evolution of macroscopic beating rate during culture; ***p < 0.0001: compared to the equivalent time in GelMA-hECTs. D) Metabolic activity of tissues, measured by Alamar blue (data was represented as normalized versus fibrin; #: compared to the equivalent time in fibrin-hECTs; p < 0.0001, *: compared to timepoints in the same hydrogel). E) Confocal IF analysis of fibrin- and GelMA-hECTs at 4 weeks, showing representative images of the hexagonal structure cellularised with cTNT^+^ hiPSC-CMs and VIM^+^ hiPSC-CFs. F) Optical mapping analysis of tissues. Fi) Action potential duration at 80% repolarization (APD_80_) for fibrin-hECTs. Fii) Calcium transient duration at 80% repolarization (CaTD_80_) for fibrin- and GelMA-hECTs (#: compared to CaTD_80_ at 0.25 and 0.5 Hz, p < 0.0001). Fiii) Response of tissues to isoproterenol. CaTD_80_ in response to isoproterenol was represented as normalized versus baseline CaTD_80_. Data shown as mean ± SEM. N ≥ 3 independent experiments, n ≥ 3. *p < 0.05; **p < 0.01; ***p < 0.001. (For interpretation of the references to color in this figure legend, the reader is referred to the Web version of this article.)

To investigate the impact of using an ECM-mimicking hydrogel in the fabricated hECTs, hiPSCs were independently differentiated to CMs and CFs per already published protocols [30]. High-purity populations were obtained, as demonstrated by the expression of cardiac troponin T (cTNT) (93.5 ± 3.02%) for hiPSC-CMs and DDR2 (97.2 ± 1.27%) for hiPSC-CFs (Fig. S1). Then, hiPSC-CMs and hiPSC-CFs were embedded in a 9:1 ratio in two different biopolymers, fibrin and GelMA, and combined with hexagonal 3D printed MEW-Scaffolds of poly(ε-caprolactone) (PCL), a pattern known to enhance cellular maturation, organization and tissue mechanical properties [27] (Fig. 1A). Based on our previous work, a high, non-limiting, and optimized seeding density of 5 × 10^6^ cells/cm^2^ was selected to promote effective cell-to-cell connectivity between hiPSC-CMs and to ensure that cell density was not a limiting factor when assessing the impact of material composition on tissue formation and function [30,54]. Constructs were maintained in culture for up to four weeks and tissue formation capacity, beating rate and structure, as well as metabolic activity and electrophysiological properties were evaluated. Contractile hECTs could be generated using both fibrin and GelMA (Fig. 1B). However, the contractile capacity for fibrin-hECTs was markedly superior, evidenced by the stronger macroscopic contraction (**Supplementary videos 1-2** for fibrin- and GelMA-hECTs at 2 weeks, respectively). A longer remodeling process was required for GelMA-hECTs, whereas fibrin-hECTs displayed an earlier coordinated beating, with homogeneous coupling of the cells throughout the tissue. This was evident already on day 1 after generation, decreasing from 60 beats per minute (bpm) to 30 bpm (0.5 Hz) by week 4. In contrast, GelMA-hECTs started to acquire a slow, localized, spontaneous contraction 48 h after tissue formation, which increased up to week 2 but decreased to 10 bpm (0.16 Hz) at week 4 (Fig. 1C). Although GelMA-hECTs achieved a beating rate equivalent to that found in fibrin at week 1 and 2 (45 bpm), in GelMA only isolated beating clusters were observed. In contrast, a homogeneous, coordinated macroscopic contraction was found on fibrin-hECTs, with bpm stabilizing after week 2 (**Supplementary videos 3-4** for fibrin- and GelMA-hECTs, respectively). In agreement with these observations, metabolic activity decreased significantly in GelMA-hECTs compared to fibrin-hECTs and declined during cultivation period (Fig. 1D). Human iPSC-CMs in fibrin constructs were well distributed throughout the scaffold, filling the hexagonal 3D structure, whereas cell and gel detachment were observed in GelMA-hECTs (Fig. 1E). These observations are in concordance with reports also showing that a GelMA hydrogel does not provide a beneficial environment for CM retention and function, necessary micropatterning necessary to provide contact guidance [19] or using cross-linking with nanomaterials to improve CM organization, maturation and contraction [21,22]. Human iPSC-derived cardiac cell viability, assessed by Live/Dead staining, did not differ between fibrin-hECTs and GelMA-hECTs after 4 weeks of culture hECTs (83.5 ± 1.75 *vs.* 85.3 ± 3.60%, respectively) (Fig. S2A–B). Nevertheless, TUNEL analysis revealed a higher proportion of apoptotic hiPSC-CMs in fibrin-hECTs compared to GelMA-hECTs (24.70 ± 4.48 *vs.* 3.91 ± 1.55%, respectively; p = 0.002) (Fig. S2C–D). This difference between cell viability methods may reflect the detection of early apoptotic events by TUNEL staining, which identifies DNA fragmentation that may precede membrane disruption, thus remaining undetected by Live/Dead assays. Notably, despite the superior metabolic activity of fibrin-hECTs, this increased apoptosis signal may be associated with active tissue remodeling processes, in which apoptosis can represent a physiological mechanism contributing to structural organization and tissue development rather than a detrimental outcome [55]. In line with this, fibrin-hECTs exhibited a significantly reduced tissue thickness compared to GelMA-hECTs (322 ± 31.1 *vs.* 1098 ± 58.3 μm, respectively; p < 0.001), which may reflect a more favorable microenvironment supporting enhanced matrix remodeling and tissue compaction (Fig. S2E–F). It is worth noting that in both cases, the thickness of the constructs is such that allows hiPSC-cardiac cells to generate contacts between pores above and below the MEW fibers, ruling out electrical isolation between pores.

Functionality of hECTs was analyzed at 4 weeks by optical mapping of action potentials (Aps) and calcium transients (CaT). Results showed that only fibrin-hECTs exhibited robust Aps with clear rate dependence, with progressive shortening of AP duration (APD) as the pacing frequency increased (0.25 – 4 Hz) and being able to respond to frequencies lower than their baseline beating (Fig. 1Fi). This is consistent with a higher level of electrophysiological maturation [56]. Importantly, APD values were in line with those described for human native tissues [57]. In contrast, none of the GelMA samples displayed detectable action potentials (N = 4, n ≥ 4). The presence of CaT in the absence of full Aps may reflect immature development of the ionic currents that normally shape the cardiac AP, as previously reported [58]. In this context, L-type calcium channels, which are crucial for initiating muscle contraction, can trigger sufficient sarcoplasmic reticulum (SR) calcium release to generate CaT, even if an AP fails to develop due to deficient key currents such as I_Na_ (fast inward sodium current) and/or I_K1_ (inward rectifier potassium current) [59]. Both hECTs showed transient changes in intracellular calcium concentration in response to electrical stimulation, but fibrin-hECTs demonstrated a more pronounced rate-dependent response in CaT duration (CaTD) shortening, whereas GelMA-hECTs showed little modulation of their CaT properties at low stimulation frequencies and were less able to shorten CaTD to match the pacing rate (Fig. 1Fii). This is in line with the very low spontaneous beating rates observed in GelMA-hECTs. Upon adrenergic stimulation with isoproterenol, a beta-adrenergic receptor agonist that increases heart rate and contractility, both fibrin- and GelMA-hECTs exhibited a prolongation of CaTD, most likely reflecting an impaired capacity for sufficiently rapid cytosolic calcium reuptake (Fig. 1Fiii). These *in vitro* findings demonstrated that fibrin-hECTs exhibited improved biological, structural, and electrophysiological properties compared to GelMA-hECTs, and highlight the relevance of using a hydrogel which allows cellular remodeling, providing an adequate mechanical environment, supporting cell adhesion and tissue organization of the hECTs.

### 3.2 Fibrin-based tissues exhibit maturation-associated transcriptomic signatures

**Fig. 2:**
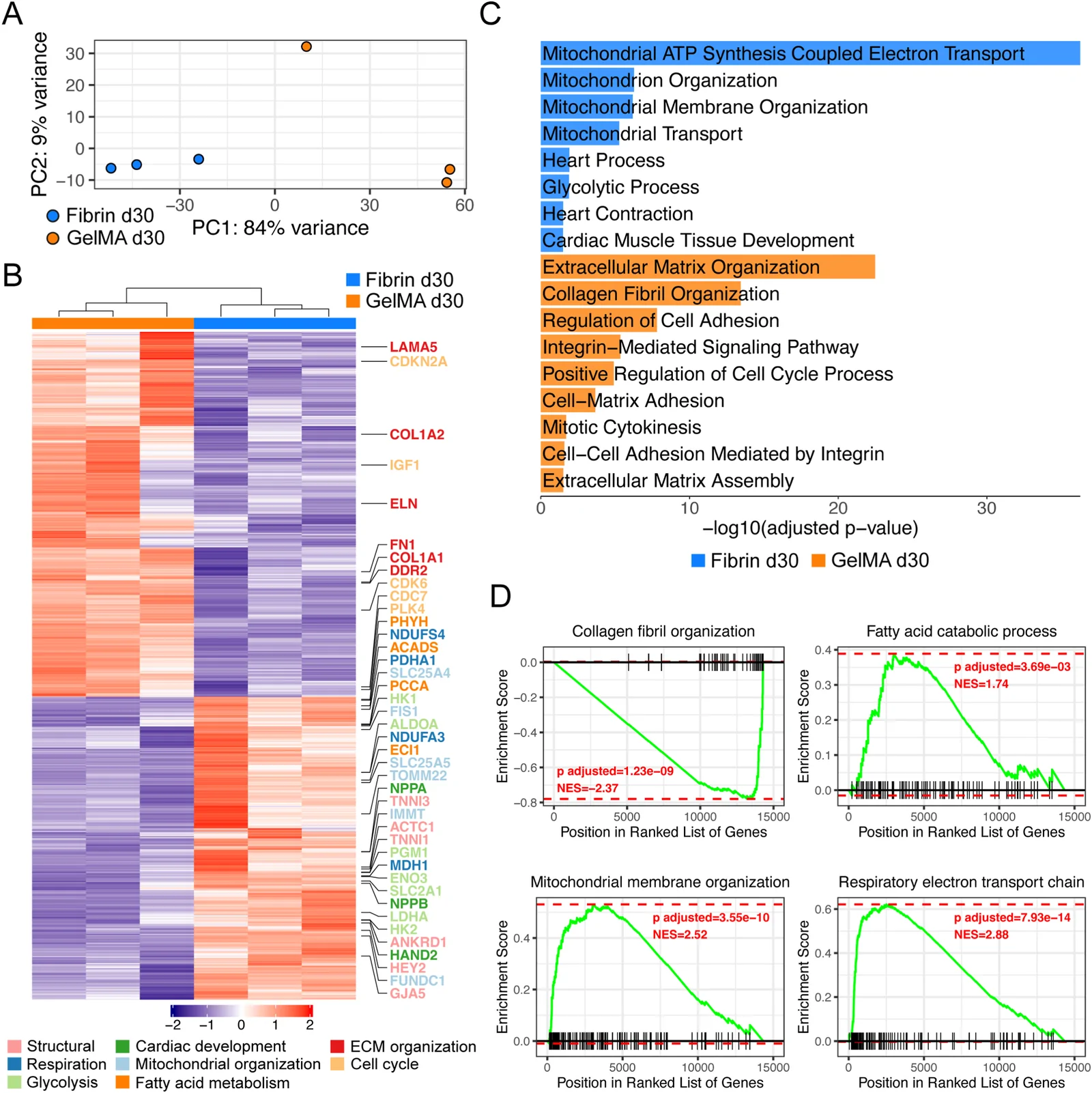
RNA-seq analysis of GelMA- and fibrin-hECTs. A) Principal component analysis. B) Heatmap of the differentially expressed genes with an absolute log2 fold-change above 0.58. Gene names are color coded by biological function. C) Gene ontology (GO) biological processes upregulated in fibrin- (blue) and GelMa-hECTs (orange) in an overrepresentation analysis. D) Gene set enrichment analysis for biological processes of interest. Positive NES indicates upregulation in fibrin-hECTs. N ≥ 3 independent experiments, n ≥ 3. (For interpretation of the references to color in this figure legend, the reader is referred to the Web version of this article.)

To analyze the influence of the biomechanical environment on the evolution and maturation of the biofabricated tissues in depth, we studied their transcriptomic profile by bulk RNA-seq at 4 weeks post-generation. As before, the hECTs consisted of hiPSC-CMs and hiPSC-CFs at a 9:1 ratio. Principal component analysis (PCA) showed a clear separation of the fibrin- and the GelMA-hECTs in the first component (PC1, 84% variance), pointing to a great impact of the biomechanical environment on the biology of the resulting hECTs (Fig. 2A). Fibrin-hECTs exhibited marked upregulation of genes and biological processes associated with cardiac structural organization, respiration, glycolysis, fatty acid metabolism and mitochondrial organization compared to GelMA-hECTs (Fig. 2B–D). The higher expression in Fibrin-hECTs could be correlated to increased energetic requirements, in line with the Alamar blue findings, key to supporting cardiac tissue development as well as a greater metabolic demand. As fetal CMs switch to oxidative phosphorylation during maturation, a concomitant increase in mitochondrial biogenesis and development of a more mature mitochondrial network is expected to take place [60,61]. In addition, *NPPA* and *NPPB* cardiac hypertrophic genes, encoding atrial and brain natriuretic peptides, were also significantly upregulated in fibrin-hECTs compared to GelMA-hECTs. These genes are highly expressed during heart development [62]. A similar increase has been observed in previous studies after the generation and maturation of hECTs, in line with the fetal-like hiPSC-derived cardiac cells undergoing maturation [12,30,35]. In contrast, GelMA-hECTs displayed a significant upregulation of genes and processes related to ECM and collagen fibril organization, which aligns with our prior observations of these constructs requiring a longer remodeling process of their extracellular environment (Fig. 2B–D). Moreover, the higher expression of genes involved in active cell cycle status in GelMA-hECTs reflects a relatively more immature phenotype of the hiPSC-CMs in the semi-synthetic environment provided by GelMA, as adult CMs undergo mitotic quiescence after maturation [63]. Overall, this comparative analysis suggests that a fibrin hydrogel provides the environment leading to the acquisition of a significantly more mature gene expression profile, as shown by a decrease in cycling-related genes, and a concomitant increase in genes related with sarcomeric organization and energy production.

**Fig. 3:**
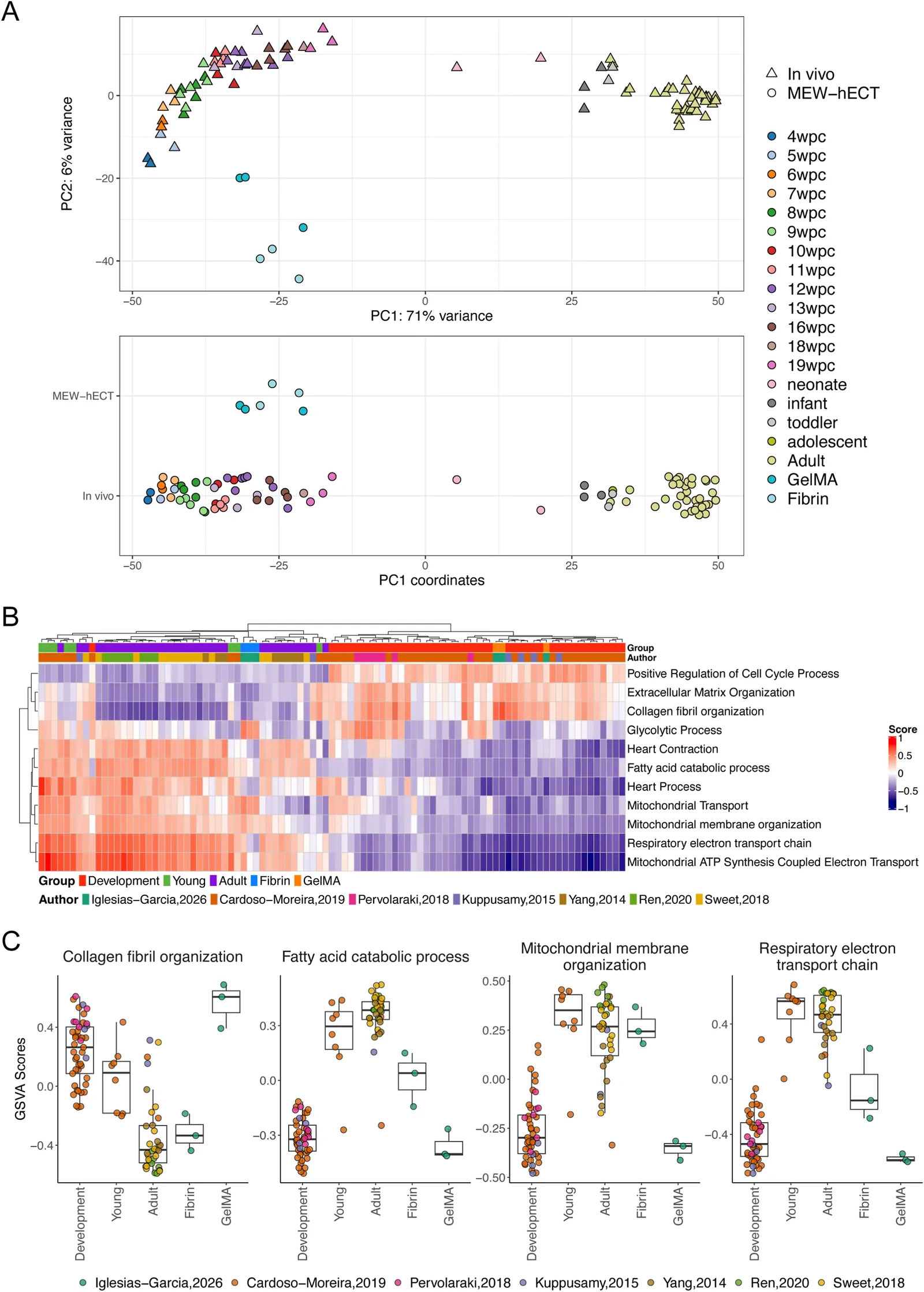
Benchmarking MEW-hECTs against human fetal and adult human myocardium. A) Principal component analysis including *in vivo* and hECT samples (top) and distribution of samples in the first principal component coordinates (bottom). Samples are color coded by detailed developmental stage. B) Heatmap of unsupervised clustered scores for differential gene sets of interest between GelMA and fibrin-based constructs. Samples are annotated by general developmental stage and publication of origin. C) Boxplots for gene set scores from the biological processes of interest represented in Fig. 2D. Scores are separated in the x-axis by developmental stage and color coded by study. Fibrin-hECTs scores have higher resemblance to adult ones, with GelMA closer to fetal samples. (For interpretation of the references to color in this figure legend, the reader is referred to the Web version of this article.)

To further assess maturation-associated features in our hECTs, we performed a comparison with publicly available fetal and adult human myocardium samples [[41], [42], [43], [44], [45], [46]]. PCA showed a clear developmental trajectory in the first component (PC1, 71% variance) with the second component (PC2, only 6% variance) separating the *in vivo* and hECT samples (Fig. 3A and Fig. S3A). As expected from the immature phenotype of hiPSC-derived cells, hECTs clustered with fetal samples in PC1, with fibrin-hECTs aligning with samples corresponding to 16 weeks post-conception (wpc) and all but one GelMA-hECT with 12-13 wpc. Nevertheless, when focusing on differential processes between both ECM-mimicking hydrogels, fibrin-hECTs had a higher resemblance to adult samples compared to GelMA-hECT, with higher gene set scores for fatty acid, mitochondrial organization and respiration-related pathways and lower scores for ECM and cell cycle processes (Fig. 3B–C, Fig. S3B and S4). In those instances where gene set scores for fibrin-hECTs were in between those for fetal and adult *in vivo* samples, they were closer than GelMA-hECTs samples to adults, except for an increased glycolytic activity, which correlates better to some fetal samples. As a result, both considering gene set scores or differentially expressed genes from those pathways, GelMA-hECTs clustered with fetal samples and fibrin-hECTs with adult and young samples (Fig. 3B and Fig. S3B), suggesting a more mature-like profile in fibrin-based constructs and pointing to an enhancement for tissue generation.

### 3.3 hiPSC-CF incorporation does not enhance tissue formation and impairs electrical properties

**Fig. 4:**
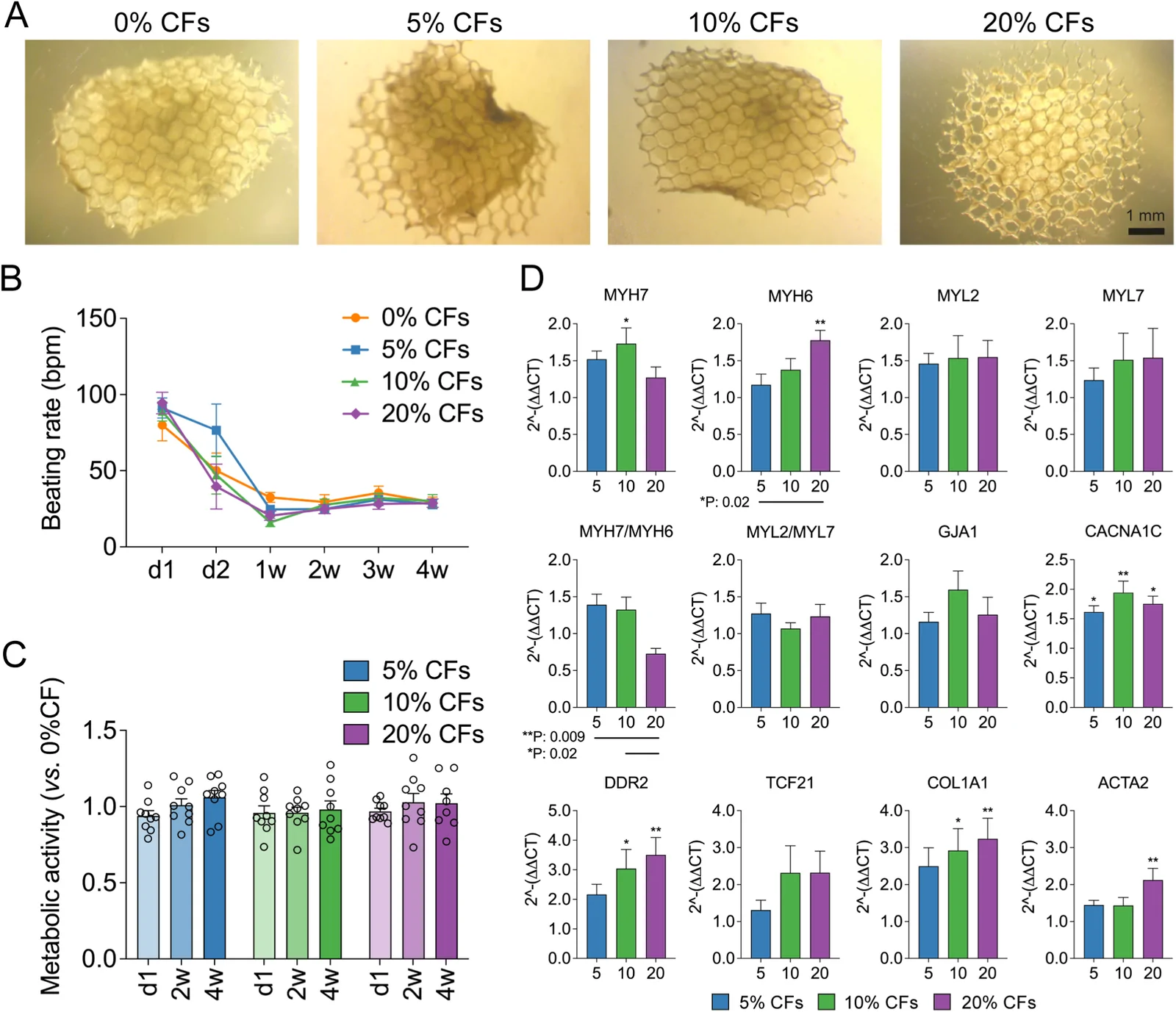
Impact of cell composition on fibrin-hECTs. A) Representative images of hECTs containing 0%, 5%, 10% and 20% of hiPSC-CFs 4 weeks after generation. B) The hECTs showed comparable beating rate, (C) as well as similar metabolic activity during the 4-week cultivation period. D) Comparative gene expression analysis between hECTs containing 0%, 5%, 10% and 20% of hiPSC-CFs at week 4. Data was normalized versus hECTs containing 0% of hiPSC-CFs in C and D. Data shown as mean ± SEM. N ≥ 3 independent experiments, n ≥ 3. *p < 0.05; **p < 0.01.

**Fig. 5:**
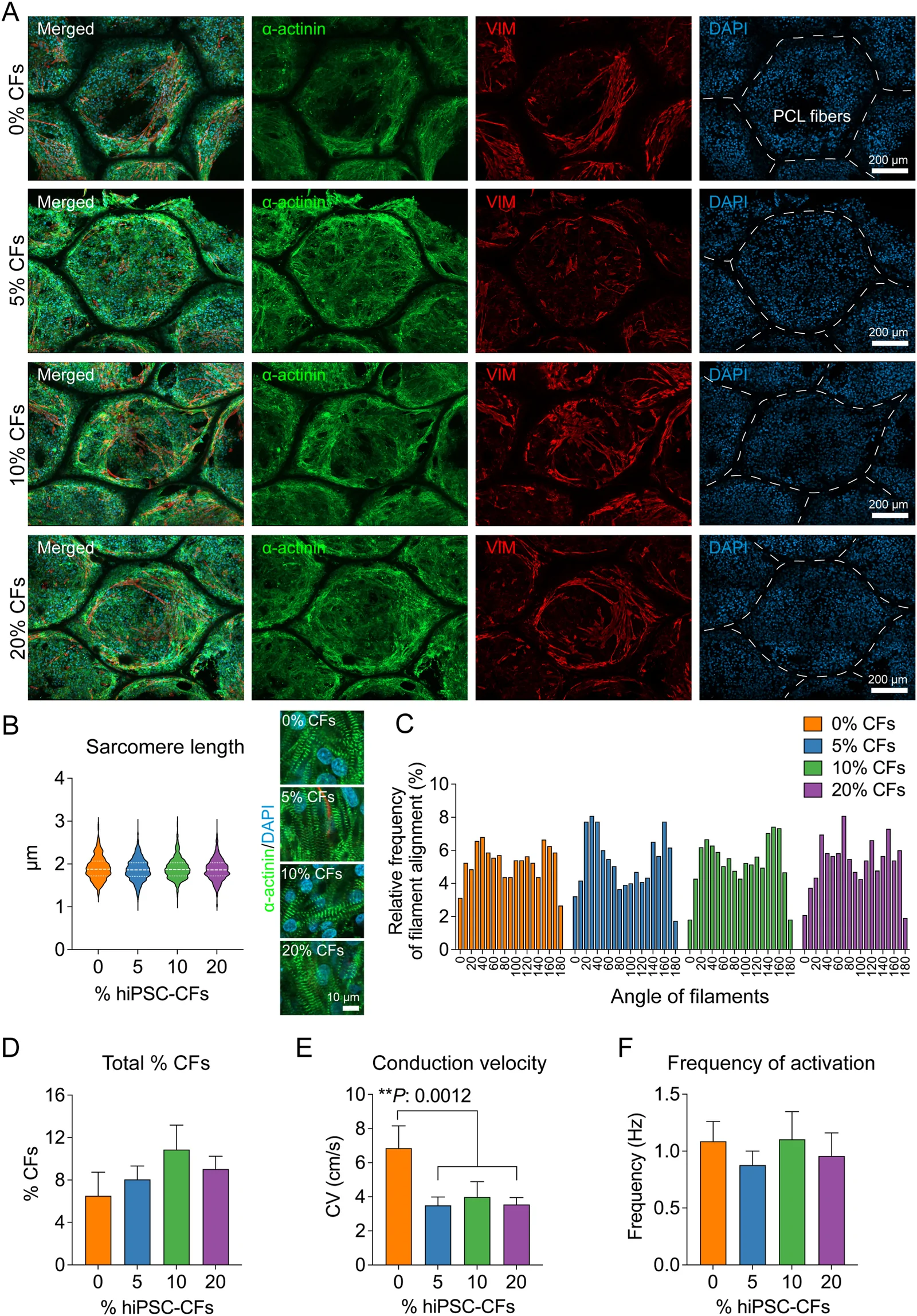
Comparative analysis of cell proportions and its impact on tissue organization and function. A) Representative confocal IF images of cell location within the hexagonal-shaped pore of the hECTs containing 0%, 5%, 10% and 20% of hiPSC-CFs, showing α-actinin + hiPSC-CMs (green) displaying well-arranged sarcomeres and VIM + hiPSC-CFs (red) within the hexagonal-shaped pore. White dashed lines indicate the area where the PCL fibers are located. Scale bars, 200 μm. B) Quantification of Z-band spacing and confocal IF images of the expression of α-actinin showing the presence of sarcomeres. Scale bars, 10 μm. C) Frequency distribution of filament alignment relative to the horizontal axis. D) Quantification of the hiPSC-CFs abundance on the hexagonal structure of the hECTs. E) Conduction velocity assessment and F) Frequency of activation varying proportions of hiPSC-CFs. Data shown as mean ± SEM. N ≥ 3 independent experiments, n ≥ 3. **p < 0.01. (For interpretation of the references to color in this figure legend, the reader is referred to the Web version of this article.)

The incorporation of stromal cells in cardiac engineered systems has been previously explored by several groups, including ours, to study their impact on cardiac tissue structure, maturation and function. However, the proportion varies widely among different platforms, ranging from 5% to 50% [31], though it is most frequently in the 10-30% range to reflect the volumetric ratio of CMs to non-myocytes in the human heart [64]. In order to determine the ideal cellular composition to generate optimal structured hECTs in our MEW-based composite system, different initial cellular proportions of hiPSC-CMs and -CFs (100:0, 95:5, 90:10 and 80:20 for CM:CF) were employed. Here, fibrin was chosen due to the previously shown superiority. Also, the number of layers in the MEW scaffolds were decreased from 35 to 20, to further refine the constructs by providing a more compliant environment. Engineered tissues were maintained in culture for up to 4 weeks and characterized by immunostaining, microscopy, metabolic activity assay, electrophysiological and gene expression analysis. In all conditions, tissues achieved macroscopic spontaneous contraction with a large enough magnitude to produce visible motion and deformation of hexagonal MEW scaffolds (Fig. 4A and **Supplementary videos 5-8** for the tissues containing 0%, 5%, 10% and 20% of hiPSC-CFs at 2 weeks, respectively). While previous works have highlighted a crucial role of non-myocytes for the fabrication of functioning tissues [5,65], here we found that fibrin-hECTs seeded only with high-purity hiPSC-CMs were also able to form stable, functioning cardiac tissues. This indicates that a better understanding is needed of (i) how stromal cells contribute to the structural organization of hiPSC-CMs in different engineered models; (ii) the extent to which MEW scaffolds can preserve the mechanical integrity of hECTs and potentially obviate the need for incorporating CFs; (iii) whether differentiation protocols produce fully functional hiPSC-CFs; and (iv) the presence of off-target fibroblasts-like populations that may influence or support tissue function. In this study, hECTs had a similar spontaneous beating rate regardless of starting cell composition, with rhythmic peaks on day 1 around 80-90 bpm, decreasing by week 1 and stabilizing from then on to around 0.5 Hz (30 bpm) (Fig. 4B). Metabolic activity was maintained during the 4 weeks in all groups (Fig. 4C). Gene expression analysis of cardiac markers by RT-qPCR showed that the expression of most CM markers remained unchanged at 4 weeks in all conditions, while the expression of CF genes *DDR2*, *COL1A1* and *ACTA2* increased significantly with the proportion of hiPSC-CFs (Fig. 4D). We observed an upregulation of the mature form of myosin heavy chain, *MYH7*, in hECTs containing 10% of hiPSC-CFs compared to those containing only hiPSC-CMs, although this increase did not lead to a significant increase in the maturity ratio *MYH7/MYH6*. The immature form of myosin heavy chain, *MYH6*, was significantly increased in tissues containing 20% of CFs compared to the other groups. This led to a marked decrease in the ratio of *MYH7/MYH6* (Fig. 4D), suggesting that the inclusion of a higher proportion of CFs in our hECTs may impair CM connectivity and tissue function. This is in agreement with a previous report where a small population of 5% adult human ventricular CFs in hECTs was identified as optimal for tissue functionality, whereas 10% and 15% disrupted electromechanical performance [66]. The Zimmermann group also demonstrated the importance of an in-depth characterization of the non-myocyte fraction for engineering force-generating cardiac tissues, but in this instance revealed that the addition of 30% human foreskin fibroblasts was optimal to promote tissue maturation [5], in contrast to the observations in the present study, suggesting that different stromal phenotypes (e.g., adult *vs.* immature hiPSC-derived) may impact tissue properties differently. Here, in all conditions, tissues displayed well distributed hiPSC-CMs throughout the hexagonal scaffold (Fig. 5A). In addition, the presence of vimentin (VIM) positive cells in hECTs formed without hiPSC-CFs (0% CF) points to the existence of a small but biologically meaningful population of off-target fibroblasts-like cells in the hiPSC-CM differentiation. This has also been reported in previous studies on engineered cardiac systems [66].

To analyze whether the inclusion of CFs could impact the arrangement of cardiac contractile fibers in our 3D system, filament structure and orientation was analyzed. Z-band spacing measured on α-actinin-stained samples and imaged by confocal microscopy at 4 weeks showed that CM sarcomere length was similar in all conditions (Fig. 5B) and close to reported values for hiPSC-CMs in other tissue engineered systems [12]. Quantification of sarcomere alignment did not differ between the different groups and in all conditions, filaments showed a tendency to align in the direction between angles 0-60° and 120-180° (Fig. 5C). This outcome may result from the lack of significant variation in the percentages of hiPSC-CFs in our system at the 4-week culture period (6.51 ± 2.22, 8.06 ± 1.26, 10.87 ± 2.30 and 9.03 ± 1.22% for the tissues initially supplied with 0%, 5%, 10% and 20% of hiPSC-CFs, respectively), which may be attributed to lower survival of hiPSC-CFs in hECTs (Fig. 5D). In contrast, a previous study showed that human CFs can provide structural guidance for CM orientation and elongation in a 2D human *in vitro* co-culture model of the myocardial environment [67]. However, higher proportions of CFs were required to affect CMs organization, ≥ 30% compared to 20% used in our study. Moreover, 2D cultures do not accurately reproduce the interactions between the cellular and extracellular environments [30].

Finally, we analyzed the electrical properties of hECTs by optical mapping of calcium transients. Tissues containing hiPSC-CFs showed a reduction in the conduction velocity (CV) compared to tissues containing only hiPSC-CMs (6.86 ± 1.31, 3.50 ± 0.49, 3.99 ± 0.90 and 3.55 ± 0.41 cm/s for the tissues containing 0%, 5%, 10% and 20% of hiPSC-CFs, respectively; p = 0.0012) (Fig. 5E), although the frequency of activation did not differ between the different groups (Fig. 5F). Similar results were reported by the Bursac laboratory, where human cardiac patches were generated with varying CM purity (48-90%) and found that patches with lower CM purity exhibited a decrease in CV [68], suggesting that the stromal environment may lead to changes in the electromechanical coupling between CMs. The CV values observed in this study are consistent with the reported range (∼2-30 cm/s) for hiPSC-CM-derived ECTs and reflects the level of functional maturation typically achieved in these systems, that more closely resemble fetal or early postnatal myocardium rather than adult myocardium (∼50-100 cm/s) [5,12,69,70]. Highly optimized systems using prolonged and progressively intensified electrical stimulation have demonstrated improved CV (∼15-25 cm/s), yet these do not reach those reported for adult myocardium [12], suggesting that further optimization, potentially through improved biofabrication strategies, is required to advance tissue maturation.

Our findings indicate that the inclusion of hiPSC-CFs in our MEW-based composite system was not beneficial for tissue functionality and in some instances, it even proved deleterious, inducing a reduction in the propagation velocity of the electrical impulse. The use of fibroblasts of different origin (hiPSC-CFs *vs*. primary cells from adult tissues) with varying proportions, together with the distinct mechanical environment provided by our composite system, could contribute to the differences between our study and others. Nevertheless, further research is needed to understand in depth the impact of fibroblasts in the engineered heart systems, not only in the formation of hECTs, but also in their structure and function.

### 3.4 hiPSC-CFs had a reduced impact on the MEW-based engineered cardiac tissues at transcriptomic level

**Fig. 6:**
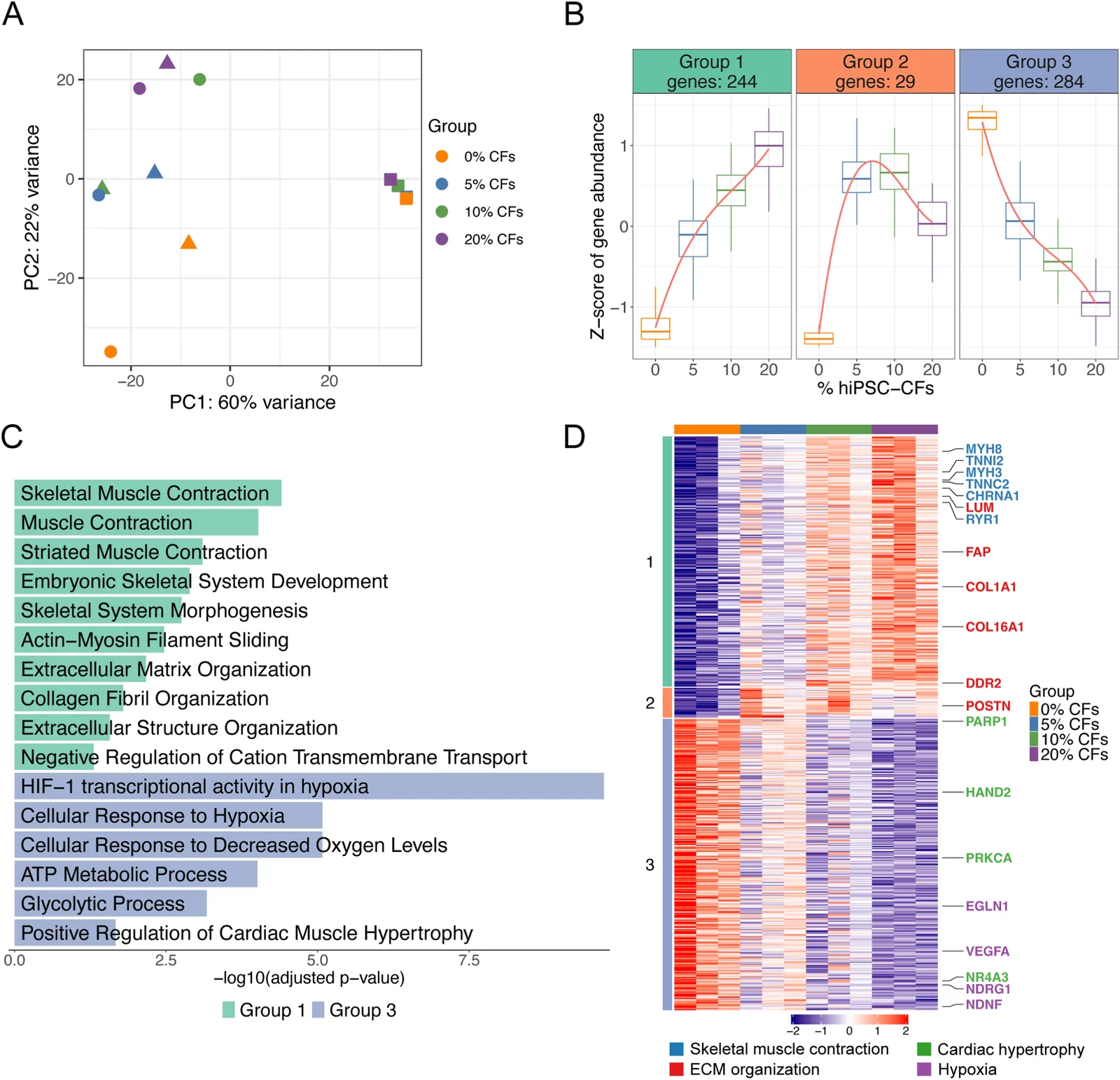
Impact of cell composition on hECTs at transcriptomic level. A) Principal component analysis (PCA). Different shapes correspond to distinct differentiations. B) Patterns of gene expression comparing tissues containing only hiPSC-CMs *vs.* those containing hiPSC-CFs. C) Gene over-representation analysis using GO biological processes database, considering the DEG behaving according to Group 1 and 3 patterns. D) Heatmap of the differentially expressed genes ordered by gene expression pattern. Gene names of interest are color coded by biological function. N ≥ 3 independent experiments, n ≥ 3. (For interpretation of the references to color in this figure legend, the reader is referred to the Web version of this article.)

To further analyze the effect of the different proportions of hiPSC-CFs in the hECTs, we also characterized them by next-generation sequencing technologies using bulk RNA-seq. Principal component analysis (PCA) showed a clear separation of the tissues containing only hiPSC-CMs from the tissues containing hiPSC-CFs in the second component (PC2) in two replicates, with the third presenting small differences among conditions. However, tissues containing hiPSC-CFs clustered together regardless of the percentage of the stromal cells (Fig. 6A). Clustering differentially expressed genes (DEG) into expression patterns revealed that the addition of higher proportions of hiPSC-CFs induced an increased expression of genes related to ECM organization and skeletal muscle contraction and a downregulation of regulation of cardiac muscle hypertrophy and cellular response to hypoxia related genes (Group 1 and 3 in Fig. 6B–D). The increased expression of genes related to collagen deposition is in line with a higher proportion of input CFs and could reflect their activation, indicating normal activity for tissue remodeling. Still, comprehensive genome-wide profiling in 3D models composed of hiPSC-CMs and hiPSC-CFs to better understand the impact of CFs on cardiac tissue organization, maturation and function remains largely unexplored (reviewed in Ref. [31]). Thus, further analysis is required to elucidate the mechanisms and cellular interactions between CFs and CMs in the 3D microenvironment. Taken together, in this study, transcriptomic analysis demonstrates that the addition of higher percentages of hiPSC-CFs in the biofabrication of hECTs can impair cardiac tissue development but their controlled inclusion could be favorable to enhance hiPSC-CM survival and inhibit hypoxia and apoptosis.

### 3.5 In silico modelling suggests increased arrhythmogenic susceptibility with hiPSC-CFs inclusion

**Fig. 7:**
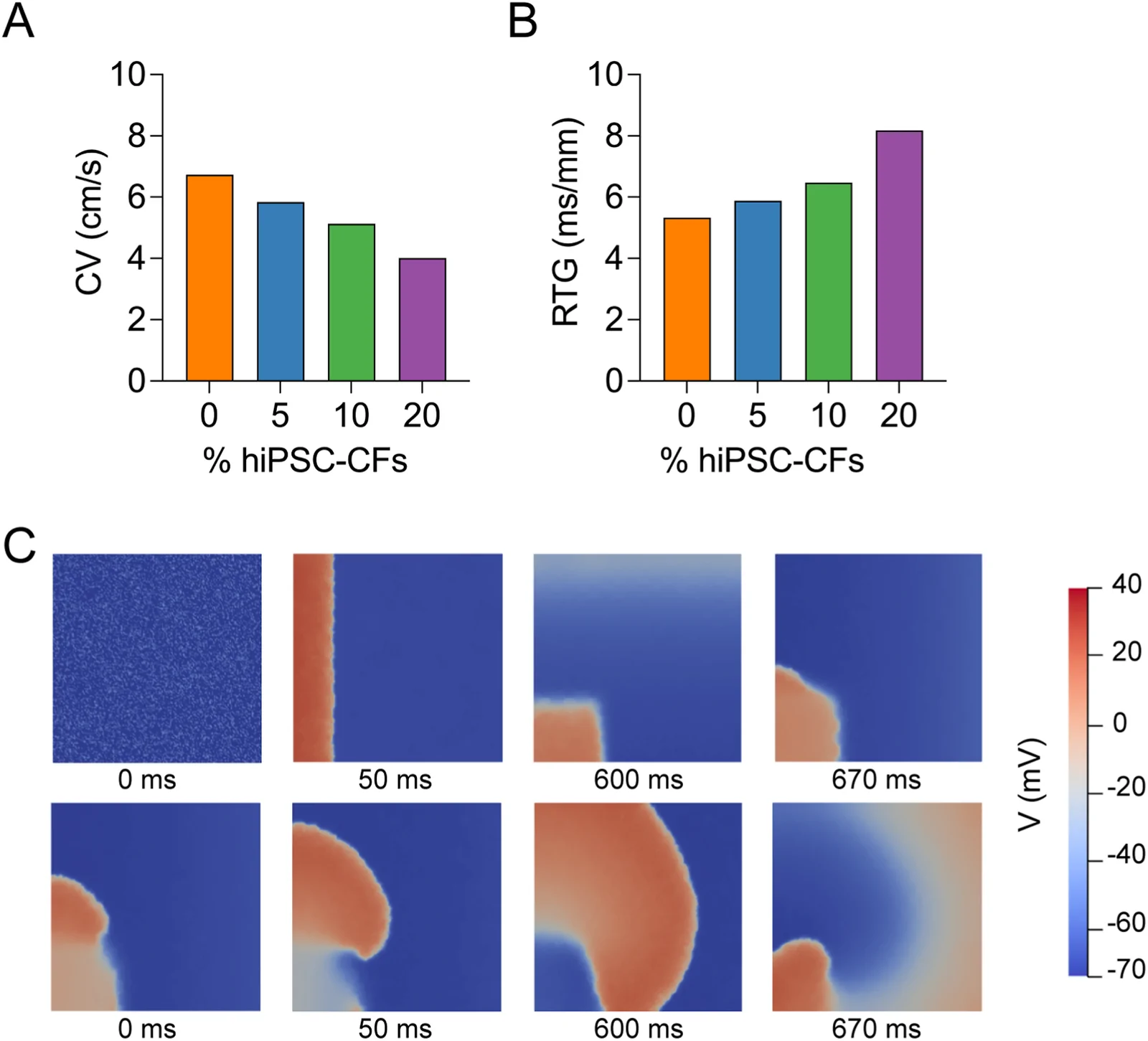
*In silico* modeling of arrhythmogenic potential of MEW-based cardiac tissues. Impact of including CFs on CV (A) and RTG (B) in simulated hECTs. C) Spiral wave generation with S1-S2 cross-stimulation protocol.

The transcriptomic upregulation of genes related to ECM organization in hECTs with increasing hiPSC-CFs abundance may result in enhanced ECM deposition, which can disrupt hiPSC-CM connectivity and impair electrical impulse propagation, ultimately increasing susceptibility to reentrant arrhythmias. Thus, we conducted a computational analysis to model how co-culturing hiPSC-CMs and -CFs influences the electrophysiological properties and arrhythmogenic potential of hECTs. Simulations using *in silico* models showed that increasing the proportion of CFs within the hECT led to a gradual reduction in CV, in agreement with experimental findings (Fig. 7A). Specifically, an 80:20 CM:CF ratio resulted in a 40% decrease in CV compared with simulated hECTs composed solely of hiPSC-CMs. To further assess repolarization dynamics, we calculated the repolarization time gradient (RTG), a measure of repolarization dispersion associated with enhanced arrhythmic susceptibility. Higher CF content was accompanied by a progressive increase in RTG (Fig. 7B). The combined reduction in CV and elevation in RTG suggested an increased likelihood for the generation and maintenance of reentrant circuits. To directly evaluate this risk, we quantified the vulnerability window (VW) length, a recognized indicator of arrhythmia propensity, using an S1-S2 cross-stimulation protocol (Fig. 7C). The presence of CFs led to a pronounced prolongation of VW length (Table 1).

**Table 1:** Effect of including CFs on VW length in simulated hECTs.

	0% CF	5% CF	10% CF	20% CF
**VW length (ms)**	20	33	35	39

It should be noted that the *in silico* models were calibrated using experimental data obtained under periodic stimulation, whereas the S1–S2 protocol was applied only computationally, therefore, reentry induction was not directly validated *in vitro*. Nonetheless, reductions in CV have been widely characterized, both computationally and experimentally, as a primary driver of increased arrhythmogenicity [71,72]. Therefore, the experimentally observed CV slowing in multi-cell-type hECTs supports the notion, inferred from our simulations, that CF incorporation enhances arrhythmogenic susceptibility in engineered constructs.

Interpretation of these computational findings in the context of native myocardium requires caution. Non-CMs constitute a substantial fraction of the heart, and the electrophysiological consequences of structural heterogeneity are not exclusively detrimental. While fibroblast clusters may reduce CV and act as anchoring sites for reentrant circuits, thereby increasing arrhythmogenic susceptibility [73], they can also exert beneficial effects under certain conditions [74]. Li et al. demonstrated that circulating traveling waves spontaneously arising in a closed-loop engineered tissue promoted sarcomeric and functional maturation of hiPSC-CMs [75]. In our engineered constructs, the predominant structural heterogeneity, stemming from the inclusion of different cell types, experimentally manifested as a reduction in CV, which proved proarrhythmic *in silico*. However, the broader biological role of non-CMs in shaping tissue electrophysiology remains complex and context-dependent.

## 4 Discussion and conclusion

We are progressing towards the rational development of hECTs by a precise assessment of the main components, aiming to recapitulate key aspects of native heart architecture. Our results highlight the relevance of the choice of the ECM-mimicking hydrogel, and provide an in-depth characterization of their differential effects upon the biology of the resulting tissues. Using a controlled MEW-based platform, we provide a detailed analysis of MEW-hECTs built with different biomaterials (fibrin, GelMA), which differ in their mechanical properties, and varying proportions of cardiac constituent cell types, which encompasses structure and tissue function assessment as well as global transcriptomic profiling.

Fibrous scaffold-based strategies have been widely explored in cardiac tissue engineering, including approaches based on electrospinning and related fabrication techniques, highlighting the importance of fibrous microenvironments in guiding CM alignment and tissue organization [76,77]. While electrospun scaffolds can provide anisotropic cues that support cardiac tissue formation and contractile activity, control over fiber orientation and manufacturing consistency is inherently limited by the fabrication process. In contrast, MEW offers a highly controlled fabrication approach that enables precise design of 3D scaffold architecture with defined fiber deposition at the micrometer scale, thereby providing a robust technology for controlled tissue organization and integration. Notably, our recent work has demonstrated that MEW-based cardiac constructs can be scaled toward clinically relevant, mechanically tunable, patch-scale architectures while maintaining structural integrity [54]. In this context, the MEW-based composite system used in this study complements existing fibrous scaffold strategies by enabling controlled evaluation of how biomaterial selection and cellular composition impact tissue organization and functional behavior in hECTs. The controlled architectural design enabled by MEW allows to generate engineered systems with improved mechanical properties, beyond what conventional bioprinting strategies can achieve [9,11,78], creating contractile directionality akin to natural myocardium [30]. However, it is plausible that doping our proposed hydrogel with more bioactive motifs such as those derived from decelullarised matrix, might help improving the maturity [79,80], which is still limited.

Integrating functional, electrophysiological, and transcriptomic analyses, the improved tissue formation observed in fibrin-hECTs is consistent with a microenvironment that allows active cell-mediated remodeling and effective transmission of biomechanical cues. Fibrin's inherent degradability and ECM-like compliance likely facilitate dynamic cell-matrix interactions, promoting maturation-associated transcriptional programs related to sarcomeric organization, and metabolic remodeling, which, together with the functional and electrophysiological outcomes, are consistent with improved functional performance. In contrast, the semi-synthetic GelMA environment displayed sustained upregulation of ECM organization, integrin-mediated signaling pathways and cell-matrix adhesion, suggesting prolonged matrix remodeling and delayed progression toward a mature CM phenotype. Nevertheless, further analyses will be required to fully define tissue maturation. In particular, future studies should include quantitative contractile force measurements, contraction and relaxation kinetics, high-resolution structural and ultrastructural analysis, detailed calcium-handling parameters and protein-level validation, as well as direct metabolic assays. Such analysis will be essential to more comprehensively define the maturation state of the constructs, determine whether the transcriptional changes identified here translate into enhanced tissue-level functionality, and help identify the cellular- and biomaterial-related factors that can guide tissue maturation.

The reduction in CV and increased arrhythmogenic vulnerability observed upon hiPSC-CFs incorporation may be explained by combined experimental and computational analyses. Transcriptomic enrichment of ECM-related genes in CF-containing tissues, together with *in silico* modeling, suggests that increased stromal content may disrupt cell-to-cell electrical coupling through enhanced matrix deposition and the increased electrical load imposed by non-excitable cells. Such alterations can slow impulse propagation, increase intracellular resistance, and enhance repolarization heterogeneity, thereby creating a substrate permissive for reentrant arrhythmias, consistent with previous *in vivo* studies [81]. Importantly, understanding the mechanisms underlying these effects requires coordinated integration of electrophysiological mapping, transcriptomic profiling, and computational modeling, rather than relying exclusively on functional assessments.

While the ordered hexagonal MEW scaffold provides robust mechanical support and promotes tissue organization, its architecture may partially compartmentalize the developing syncytium and thereby limit direct lateral electrical coupling between adjacent regions. Thus, the conduction velocities measured here likely reflect both the still-immature state typical of hiPSC-CM engineered tissues and the balance imposed by the current scaffold design between structural guidance and electrical continuity. Future studies will evaluate MEW architectures with staggered layer offsets and through-thickness interconnections to enhance inter-pore coupling while preserving tissue organization [82,83].

Overall, this work provides system-specific conceptual insight demonstrating that the biomechanical environment defined by the hydrogel, together with cellular composition, influence the organization and functional behavior of MEW-based engineered human cardiac tissues. Through an integrated multimodal analysis, this study offers a framework for evaluating biomaterial and components within this system, identifying fibrin as a more supporting hydrogel than GelMA under the conditions tested and highlighting the context-dependent impact of hiPSC-CF inclusion. While additional further analysis will be required to fully define tissue maturation, these findings provide a foundation for future optimization of biomimetic hECTs, including subsequent incorporation of vascularization and tissue integration strategies.

## CRediT authorship contribution statement

**Olalla Iglesias-García:** Writing – review & editing, Writing – original draft, Visualization, Validation, Methodology, Investigation, Funding acquisition, Formal analysis, Data curation. **Asier Ullate-Agote:** Writing – review & editing, Writing – original draft, Software, Methodology, Investigation, Formal analysis, Data curation. **Jiabin Qin:** Writing – review & editing, Methodology, Investigation. **Aida Oliván-Viguera:** Writing – review & editing, Software, Methodology, Investigation, Formal analysis, Data curation. **Laura García-Mendívil:** Writing – review & editing, Software, Methodology, Investigation, Formal analysis, Data curation. **Gerardo Cedillo-Servin:** Methodology, Investigation. **Johannes Braig:** Writing – review & editing, Methodology, Investigation. **Jose Valdés-Fernández:** Software, Methodology, Investigation. **Inge Dokter:** Methodology, Investigation. **Ilazki Anaut-Lusar:** Methodology, Investigation. **Eduardo Larequi:** Methodology, Investigation. **Patxi San Martin-Uriz:** Methodology, Investigation, Data curation. **Paula Aguirre-Ruiz:** Methodology, Investigation, Data curation. **Pedro Vicente:** Writing – review & editing, Methodology, Investigation. **Ricardo M. Rosales:** Methodology, Investigation, Data curation. **Ana María Sánchez de la Nava:** Methodology, Investigation, Formal analysis, Data curation. **Ainitze Gereka Goienetxe:** Writing – review & editing, Methodology, Investigation. **Ane Miren Zaldua:** Writing – review & editing, Supervision. **María Eugenia Fernández-Santos:** Writing – review & editing, Validation, Supervision. **Manuel García de Yébenes:** Writing – review & editing, Validation, Supervision. **Juan José Gavira:** Writing – review & editing, Validation, Supervision. **Miguel Castilho:** Writing – review & editing, Validation, Supervision. **Paula M. Alves:** Writing – review & editing, Validation, Supervision. **Margarida Serra:** Writing – review & editing, Validation, Supervision. **Ming Wu:** Methodology, Investigation. **Stefan Janssens:** Writing – review & editing, Validation, Supervision. **Tomasz Jüngst:** Writing – review & editing, Validation, Supervision. **Jürgen Groll:** Writing – review & editing, Validation, Supervision. **Jos Malda:** Writing – review & editing, Validation, Supervision. **Esther Pueyo:** Writing – review & editing, Validation, Supervision, Data curation. **Manuel Doblaré:** Writing – review & editing, Validation, Supervision. **Joost P.G. Sluijter:** Writing – review & editing, Validation, Supervision. **Alain van Mil:** Writing – review & editing, Validation, Supervision, Investigation, Data curation. **Felipe Prósper:** Writing – review & editing, Supervision, Resources, Project administration, Funding acquisition, Conceptualization. **Manuel M. Mazo Vega:** Writing – review & editing, Writing – original draft, Visualization, Validation, Supervision, Resources, Project administration, Investigation, Funding acquisition, Conceptualization.

## Funding

This work was supported by the European Union NextGenerationEU/PRTR; the H2020 research and innovation programme under grant agreements No 874827 (BRAVƎ) and by the Ministerio de Ciencia, Innovación y Universidades CARDIOPRINT (PLEC2021-008127), VOLVAD (PID2022-142562OB-I00) and INVESTTRA (PID2022-142807OA-I00) funded by MICIU/AEI /10.13039/501100011033; Gobierno de Navarra Proyectos Estratégicos IMPRIMED (0011-1411-2021-000096) and BIOHEART (0011-1411-2022-000071) and Gobierno de Navarra Proyectos Colaborativos BIOGEN (PC020-021-022). This work was also supported by the Research Unit UID/04462: iNOVA4Health – Programme in Translational Medicine, financially supported by Fundação para a Ciência e Tecnologia / Ministério da Educação, Ciência e Inovação and the Associate Laboratory LS4FUTURE (LA/P/0087/2020). A.U.A. is the recipient of a Miguel Servet contract (CP25/00020) funded by the Instituto Carlos III (ISCIII) and co-funded by the European Union.

## Declaration of competing interest

The authors declare that they have no known competing financial interests or personal relationships that could have appeared to influence the work reported in this paper.

## Data Availability

The data that support the findings of this study are available from the corresponding author upon reasonable request. The RNA-seq data generated in this study have been deposited in the NCBI Gene Expression Omnibus (GEO) repository (https://www.ncbi.nlm.nih.gov/geo/) under the accession numbers GSE315963 (fibrin and GelMA hydrogel comparison) and GSE315964 (hiPSC-CFs proportions)
