# Supplementary material for: A study on extracellular and cellular composition to advance towards the rational design of contractile human myocardium
Source: Mater Today Bio. 2026 Sep 4;40:103647. doi: 10.1016/j.mtbio.2026.103647 (PMC13577836; doi:10.1016/j.mtbio.2026.103647)
Supplement: Supplementary file 1 [file mmc2.docx]

**Supplementary data**

**Fig. S1.** Cardiac differentiation potential of hiPSCs. A) Schematics of the hiPSC-CM differentiation process, including metabolic selection, leading to highly pure cultures (B) >90% by FACS for cardiac troponin T (cTNT) (N=3). C) Bright field image of the cell morphology at day 20 of differentiation is shown. D) Cardiomyocytes were selected at day 22 of differentiation and stained for α-actinin (Alexa Fluor 488; green), demonstrating a strong presence of sarcomeres. E) Schematics of the hiPSC-CF differentiation process, occurring through an intermediate epicardial stage, leading to highly pure cultures (F), as assessed by FACS for DDR2 (>90%; N=3). G) Transmitted light microscopy images of differentiated cells on day 17. H) Confocal IF image of the expression of DDR2 (Alexa Fluor 488; green). Nuclear staining was performed with DAPI. Scale bars: 100 μm (C, G), 20 μm (D, H).

**Fig. S2.** hiPSC-CMs viability and histological analysis of MEW-hECTs. Representative images for A) Live/Dead staining, C) TUNEL assay combined with immunofluorescence for cTnT, and E) Hematoxylin-Eosin staining. (B, D and F) Quantitative analysis of staining and histological parameters. Scale bars: 50 μm (A), 20 μm (C), 0.5 mm (Fibrin) and 1 mm (GelMA) (E). Data are represented as mean ± SEM (N=3, n=3). **p<0.01, ***p<0.001 (unpaired *t*-test).

**Fig. S3.** Comparison between MEW-hECTs and human fetal and adult human myocardium. A) Principal component analysis including *in vivo* and hECT samples, color coded by study of origin and shaped according to general developmental stage. B) Heatmap of scaled expression of the genes of interest highlighted in Fig. 2B for *in vivo* and hECTs samples, with unsupervised clustering. Gene names are color coded by biological function. Samples are annotated by general developmental stage and publication of origin.

**Fig. S4.** Comparison between MEW-hECTs and human fetal and adult human myocardium. Boxplots for gene set scores from biological processes of interest of the overrepresentation analysis represented in Fig 2C covering the different biological functions. Scores are separated in the x-axis by developmental stage and color coded by study. The scores are also represented in Fig. 3B heatmap.
